# Supplementary material for: UM-164, a Dual Inhibitor of c-Src and p38 MAPK, Suppresses Proliferation of Glioma by Reducing YAP Activity
Source: Cancers (Basel). 2022 Oct 29;14(21):5343. doi: 10.3390/cancers14215343 (PMC9658092; doi:10.3390/cancers14215343)
Supplement: Supplementary file 1 [file cancers-14-05343-s001.zip › Figures S1-S3, Table S1.pdf]

Supplementary file

Table S1

Antibodies	Source	Identifier
Cyclin D1	Cell Signaling Technology	2922S
CDK2	Santa Cruz Biotechnology	sc-6248
CDC6	Santa Cruz Biotechnology	sc-9964
p-Src416	Cell Signaling Technology	59548
Src	Cell Signaling Technology	2108S
p-p38	Cell Signaling Technology	9215S
p38	Cell Signaling Technology	9212S
p-YAP127	Cell Signaling Technology	4911S
p-YAP397	Cell Signaling Technology	13619S
YAP1	NOVUS	NB110-58358
AXL	Cell Signaling Technology	8861S
CYR61	Santa Cruz Biotechnology	sc-374129
V5-tag	Cell Signaling Technology	13202
Flag-tag	Proteintech	20543-1-AP
Ki67	Abcam	ab16667
GAPDH	HuaBio	ET1601-4
LAMIN A/C	Abcam	ab133256
HRP Conjugated Goat anti-Mouse IgG	HuaBio	HA1006
HRP Conjugated Goat anti-Rabbit IgG	HuaBio	HA1001
Alexa Fluor 488 goat anti rabbit	Invitrogen	A11034

Figure S1

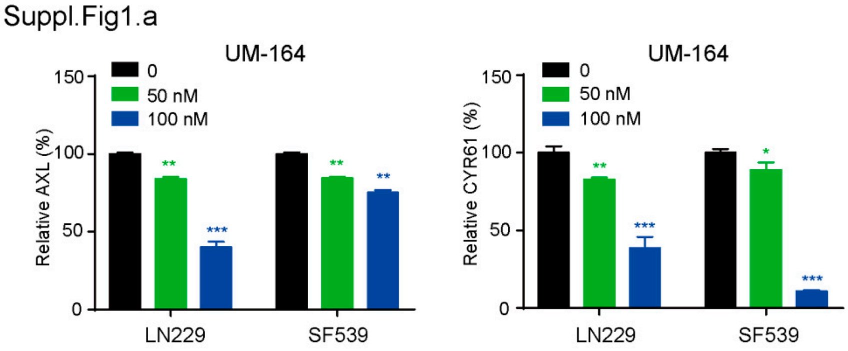

Suppl. Figure 1

(a) LN229 and SF539 cell lines were treated with Dasatinib and UM-164. The protein levels of AXL and CYR61 corresponding to GAPDH were quantified and analyzed. *$p < 0.05$, **$p < 0.01$, ***$p < 0.001$.

Figure S2

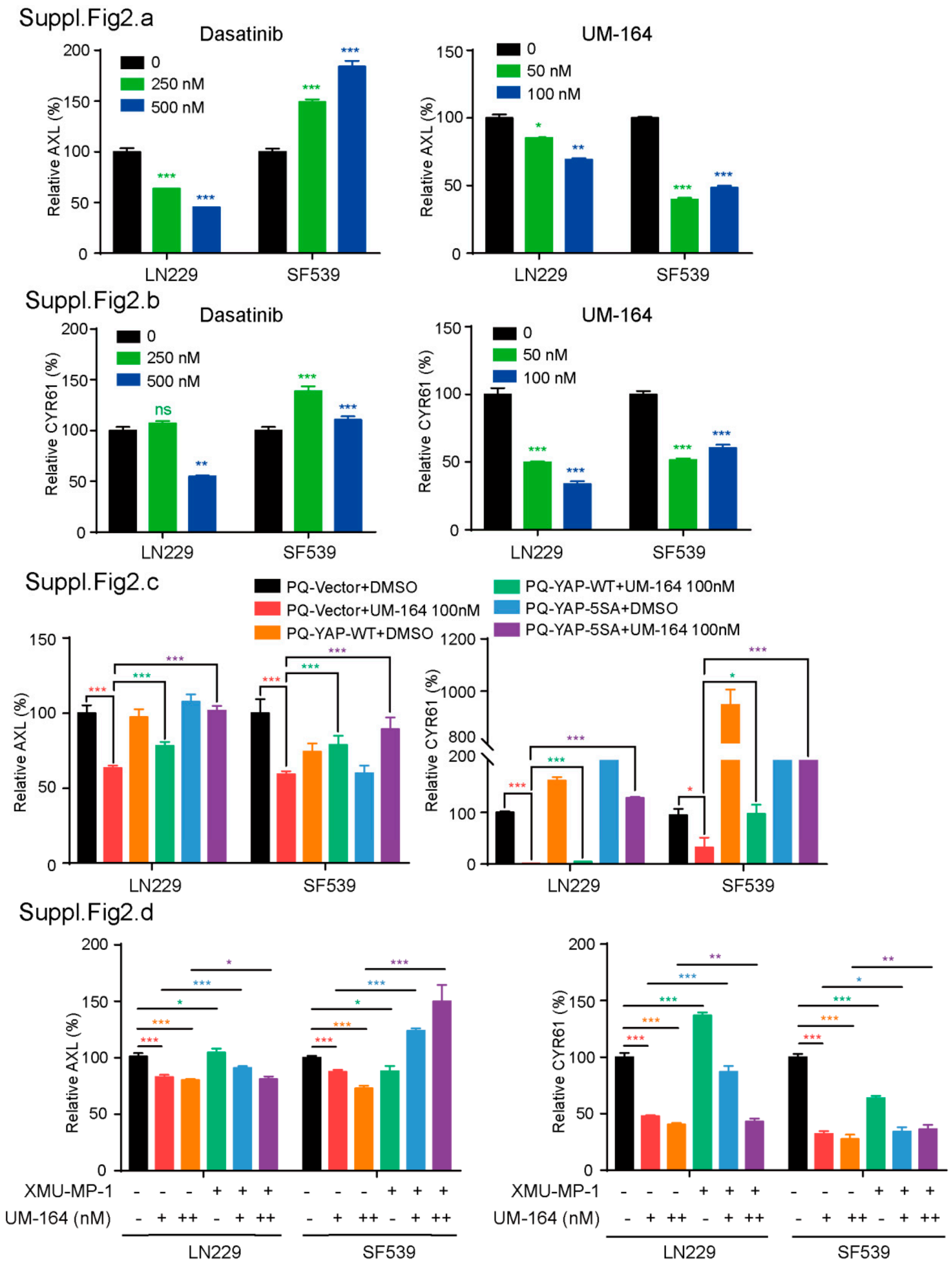

Suppl. Figure S2

(a, b) The protein levels of AXL and CYR61 corresponding to GAPDH in LN229 and SF539 cell lines incubated with Dasatinib and UM-164 were quantified and analyzed.

(c) LN229 and SF539 cells transfected with PQ-vector, YAP-WT or YAP-5SA were treated with vehicle or 100 nM UM-164. The protein levels of AXL and CYR61 corresponding to GAPDH were calculated and analyzed.

(d) LN229 and SF539 cells were pretreated with 1 μM XMU-MP-1, and then treated with vehicle or 100 nM UM-164 for 24 h. The protein

levels of AXL and CYR61 corresponding to GAPDH were calculated and analyzed. *p < 0.05, **p < 0.01, ***p < 0.001.

Fig.S3

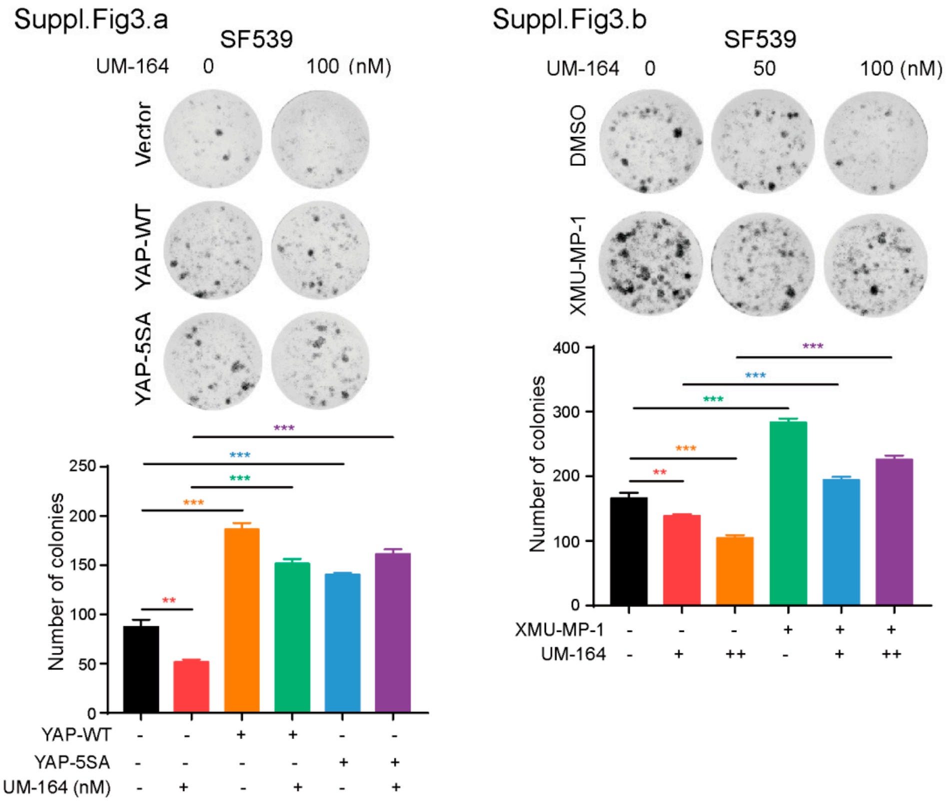

Suppl. Figure S3

(a) Colony formation assays on SF539 cells were performed after the infection of YAP-WT and YAP-5SA into the cells exposed to UM-164. Number of colonies were measured.

(b) Colony formation assays on SF539 cells were performed after the incubation with XMU-MP-1 and UM-164. Number of colonies were measured. **p < 0.01, ***p < 0.001.
