# Supplementary figures and images for: UM-164, a Dual Inhibitor of c-Src and p38 MAPK, Suppresses Proliferation of Glioma by Reducing YAP Activity
Source: Cancers (Basel). 2022 Oct 29;14(21):5343. doi: 10.3390/cancers14215343 (PMC9658092; doi:10.3390/cancers14215343)

Fig.2B

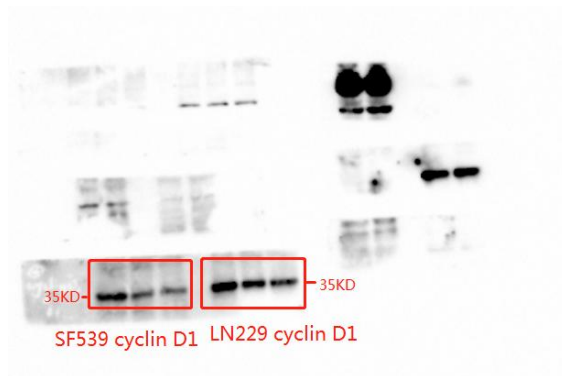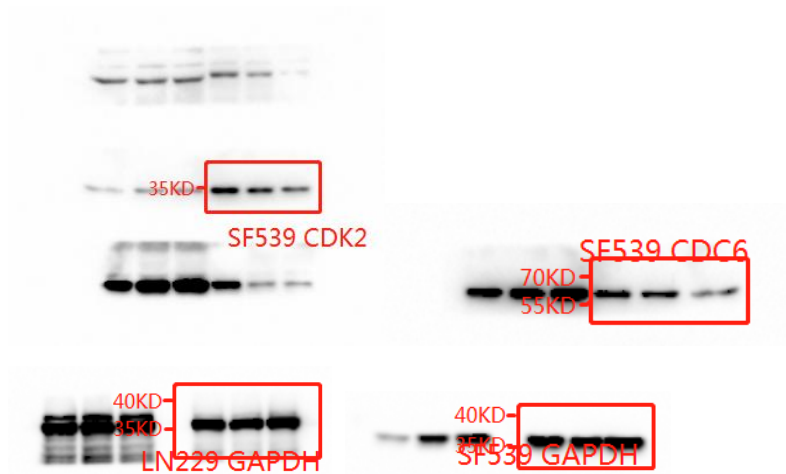

Fig.2D

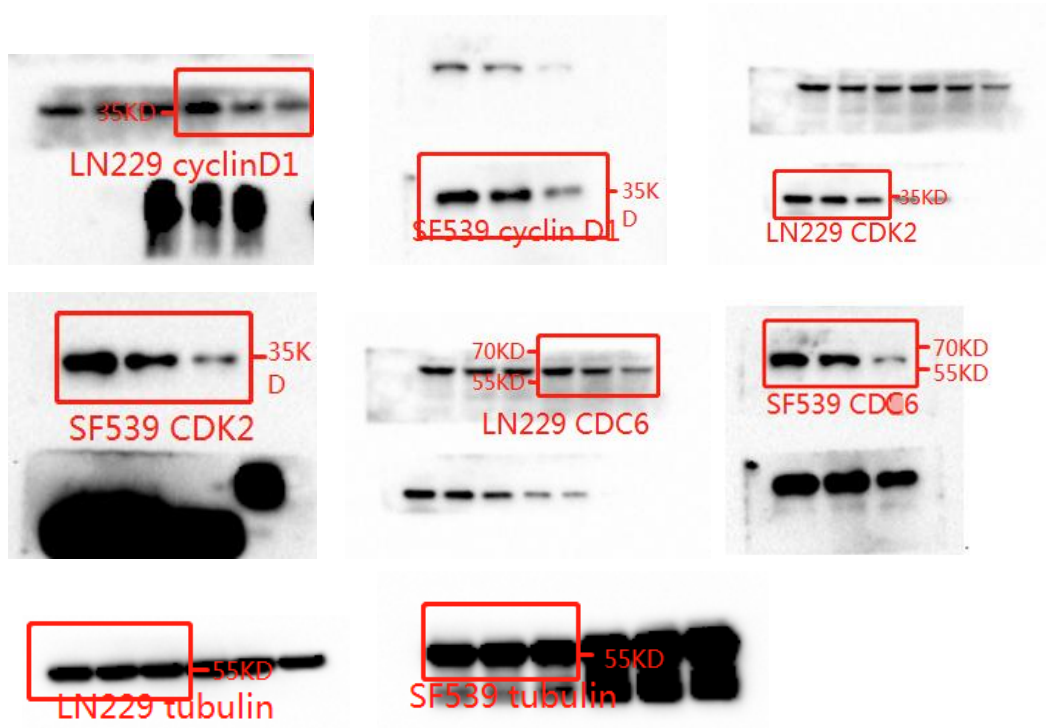

Fig.3A

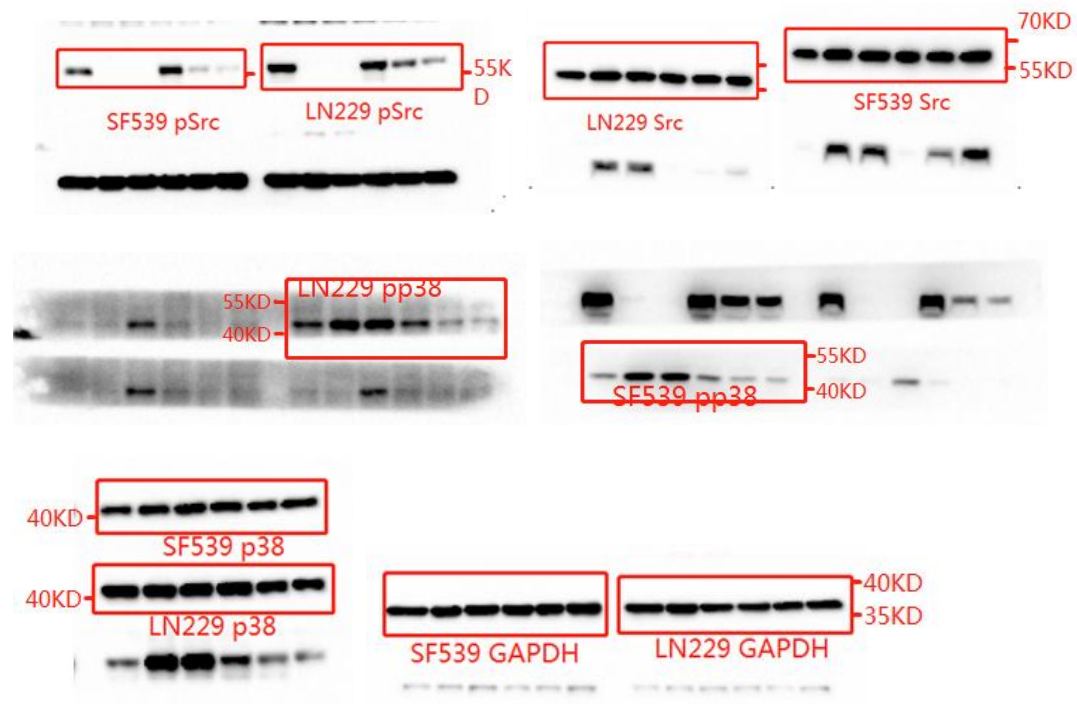

Fig.3D

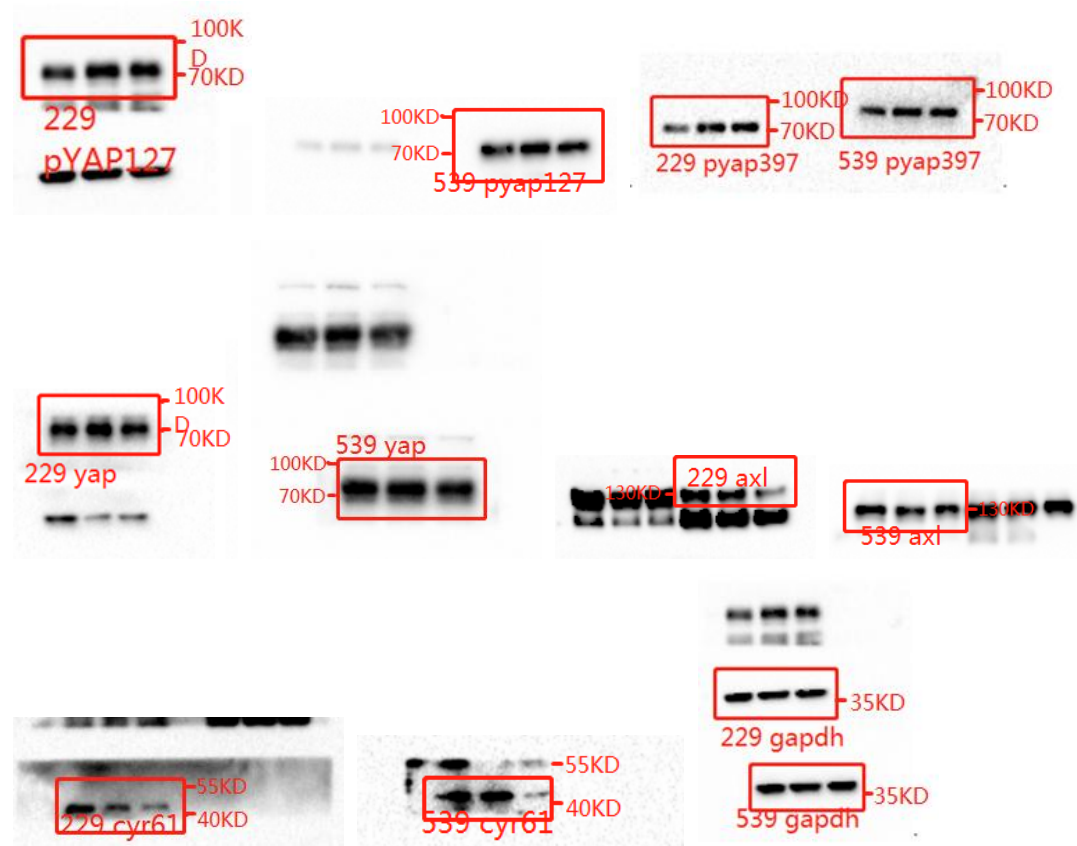

Fig.3F

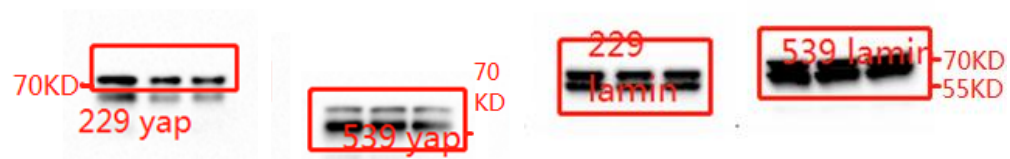

Fig.4A

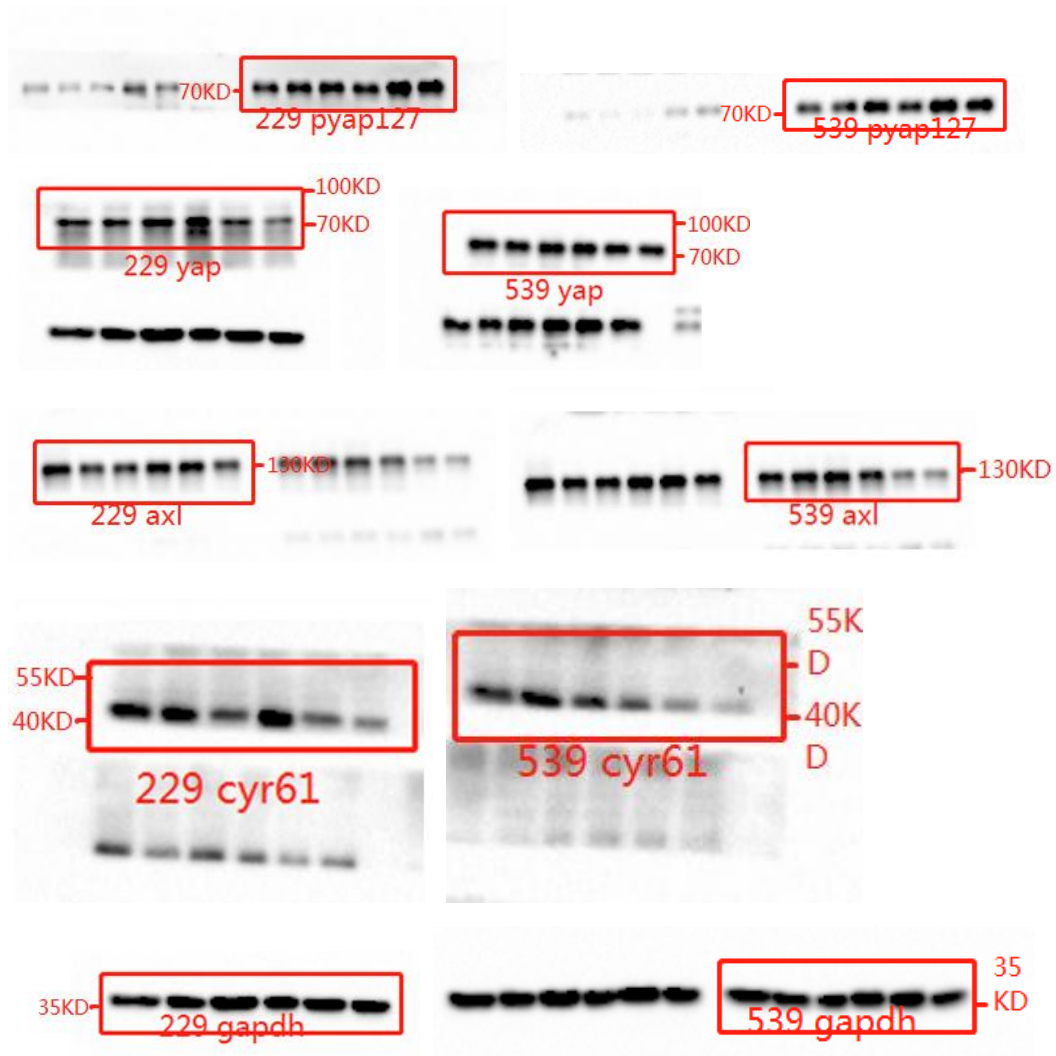

Fig.4B

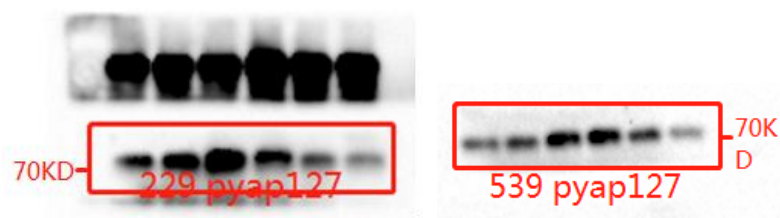

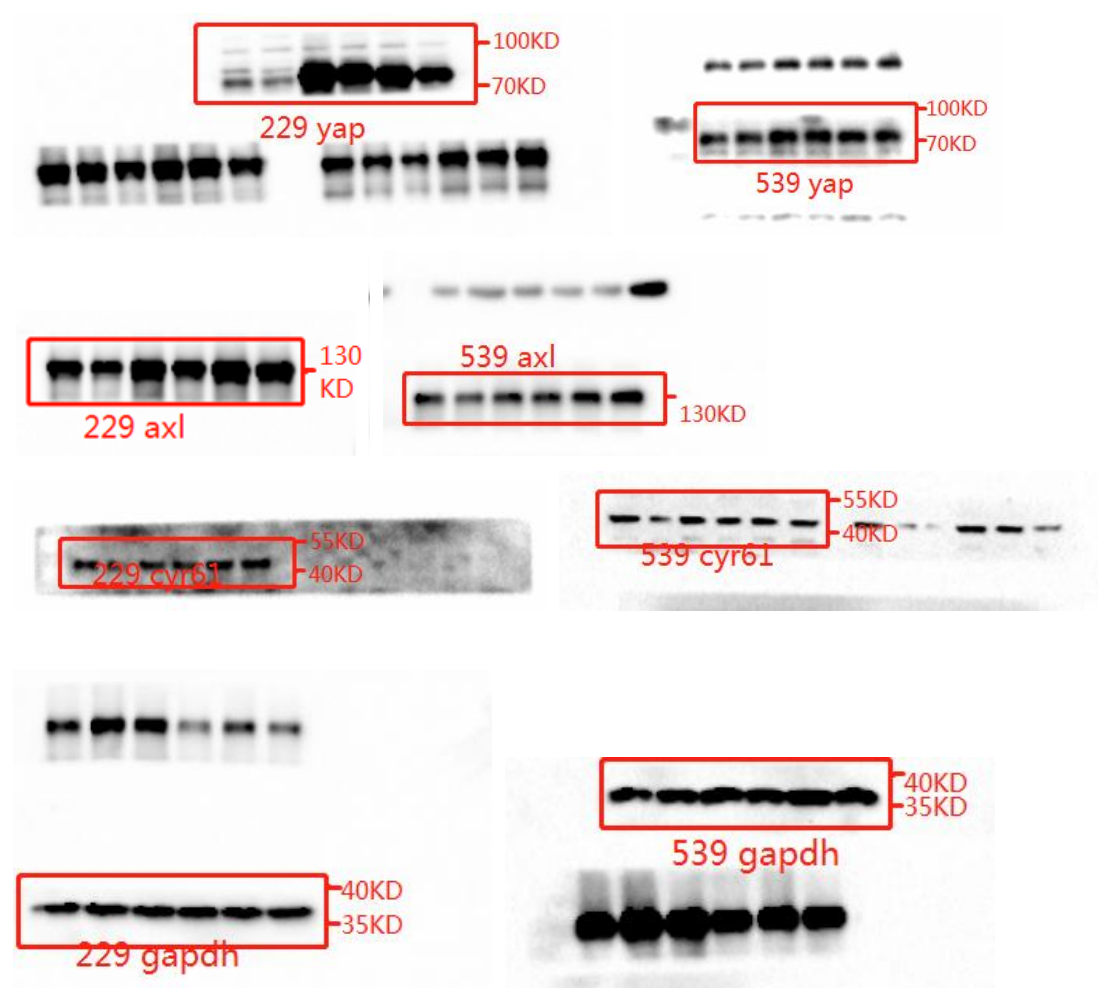

Fig.4D

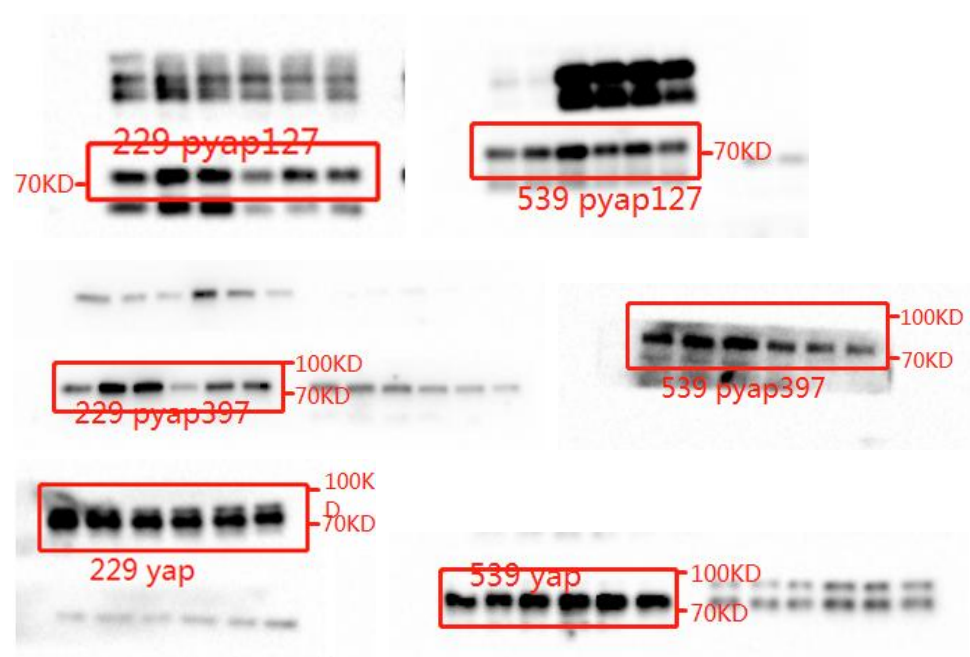

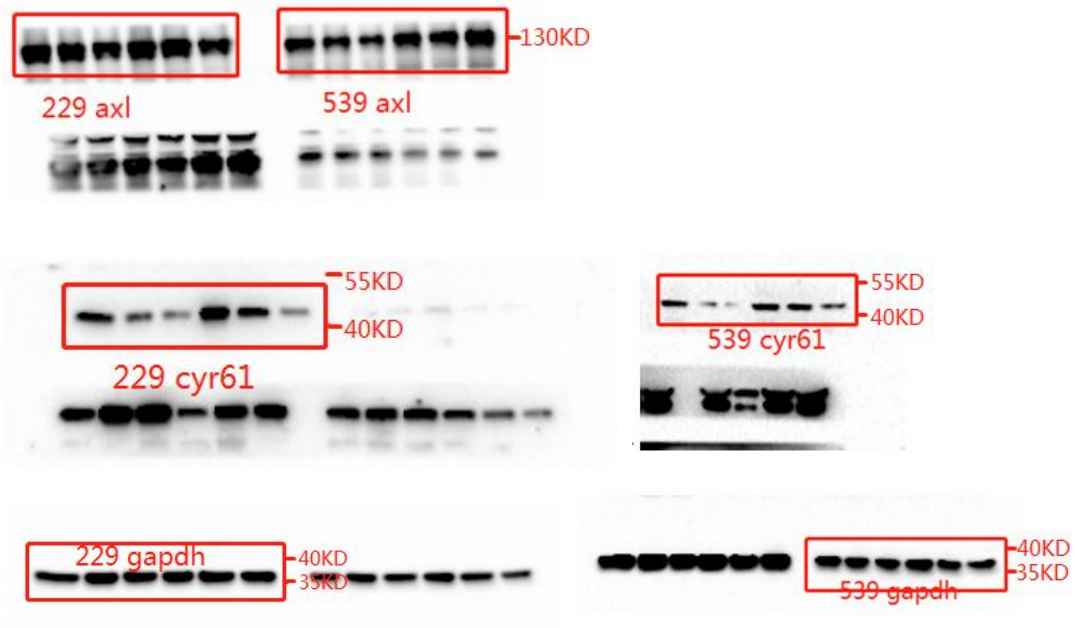

Fig.5A

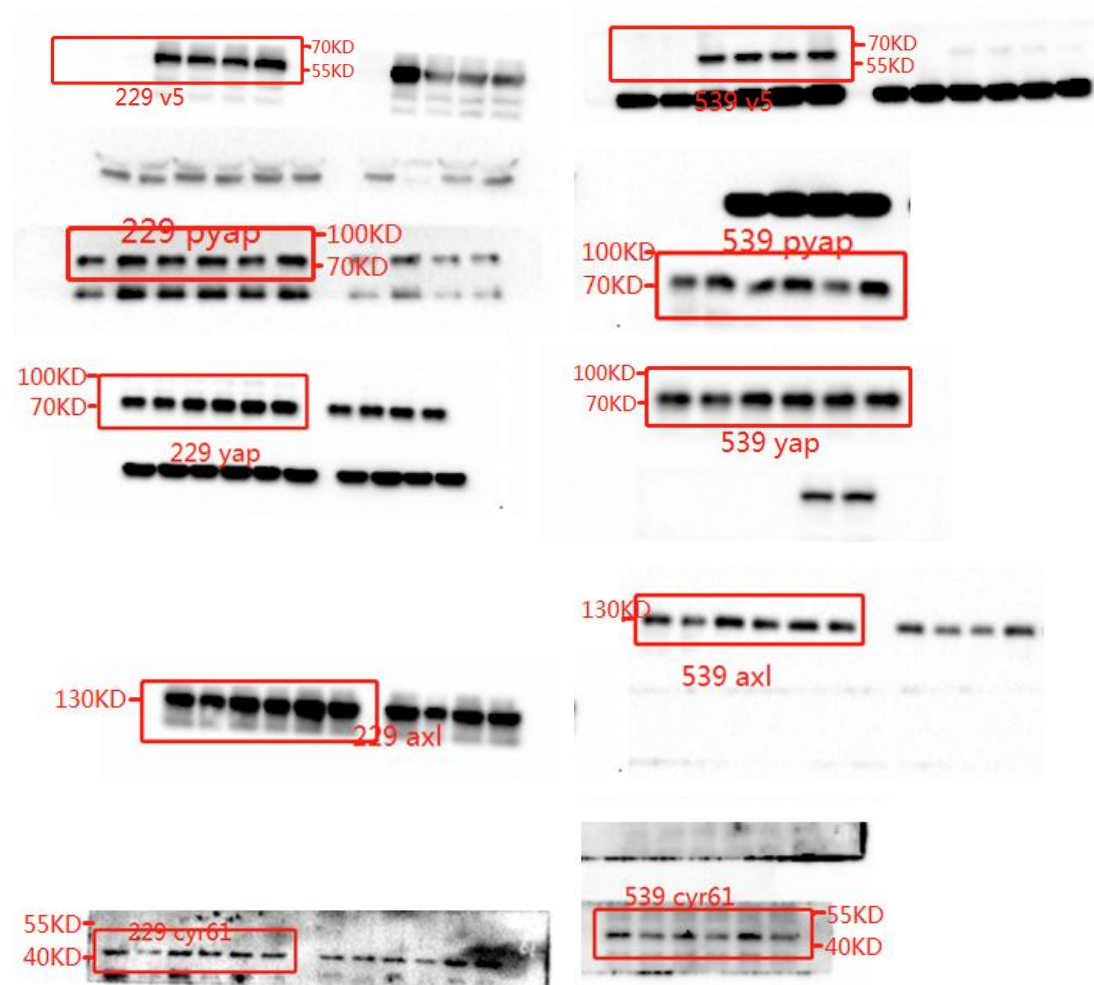

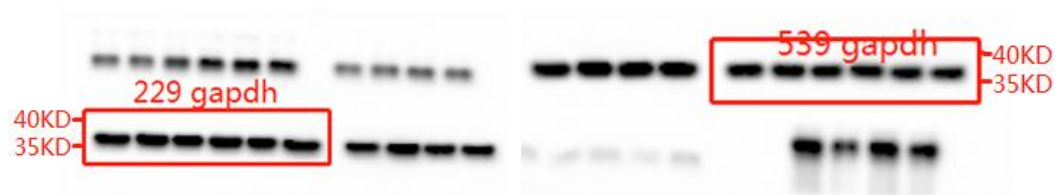

Fig.5B

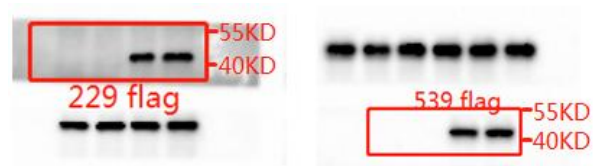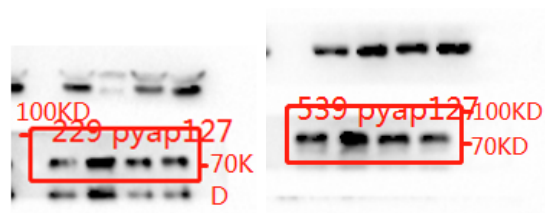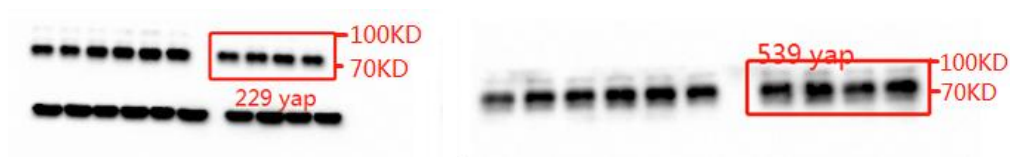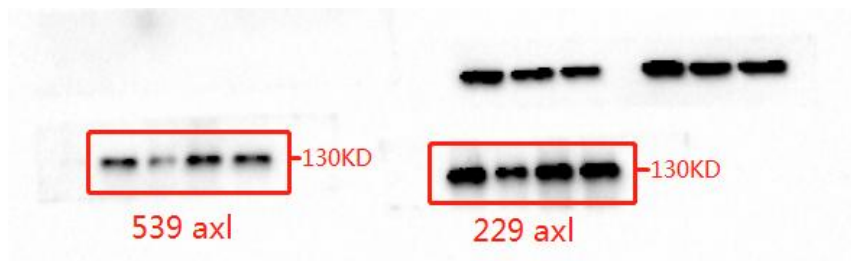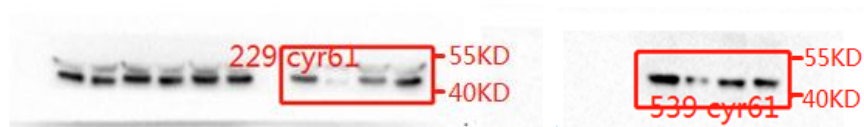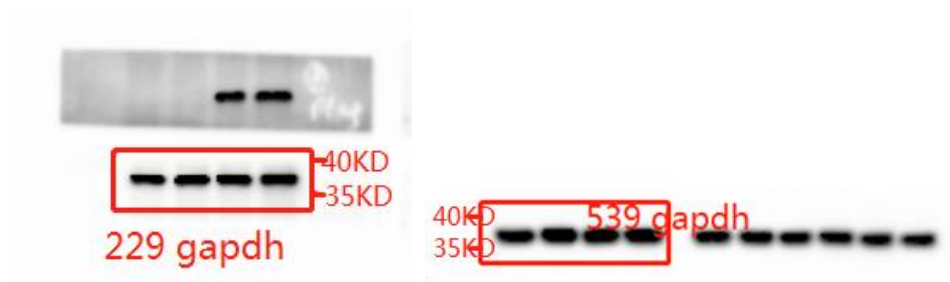

Fig.6E

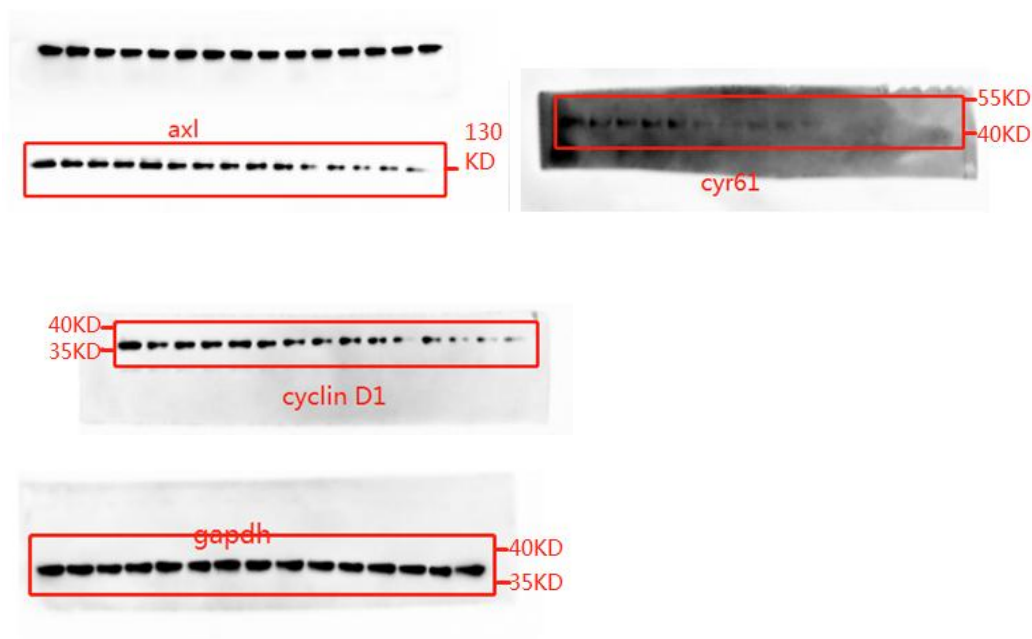

Supplement: Supplementary file 1 [file cancers-14-05343-s001.zip › File S1.pdf]
